# Elastic Characterization of Acrylate-Based Liquid Crystal Elastomers

**DOI:** 10.3390/polym17050614

**Published:** 2025-02-25

**Authors:** Gevorg S. Gevorgyan, Maksim L. Sargsyan, Mariam R. Hakobyan, Matthew Reynolds, Helen F. Gleeson, Rafik S. Hakobyan

**Affiliations:** 1 Institute of Physics, Yerevan State University, Yerevan 0025, Armenia; g.gevorgyan@ysu.am (G.S.G.); mariam.hakobyan.rafik@gmail.com (M.R.H.); rhakob@ysu.am (R.S.H.); 2 School of Physics and Astronomy, University of Leeds, Leeds LS2 9JT, UK; m.reynolds@leeds.ac.uk (M.R.); h.f.gleeson@leeds.ac.uk (H.F.G.)

**Keywords:** liquid crystal elastomers, elastic anisotropy, mechanical deformation, elastic constants

## Abstract

Liquid crystal elastomers (LCEs) are innovative materials best known for their reversible shape and optical property changes in response to external stimuli such as heat, light, and mechanical forces. These unique features position them as promising candidates for applications in emerging technologies. The determination of the mechanical properties of these materials is important for the study of the interaction between orientational and mechanical deformations of LCEs. Importantly, thoroughly characterizing the mechanical and elastic properties of LCEs is essential for their efficient design and integration into various devices. In this study, a full elastic characterization of promising acrylate-based LCE materials that are auxetic above a material-dependent strain threshold (~0.4 for the material studied here) was carried out. Highly aligned macroscopic samples were fabricated, allowing us to determine, for the first time, the five elasticity coefficients that enter into the elastic-free energy density of acrylate-based LCE materials, as well as the Young’s moduli and Poisson ratios. Our approach involves connecting measured strains with elasticity coefficients and using data obtained from three tensile experiments. Specifically, the measured Young’s moduli are on the order of MPa, with an anisotropy ratio (E‖/E⊥) of ~4.5. Moreover, the longitudinal Poisson ratios are both close to 0.5, confirming a uniaxial elastic response at low strains in these LCE samples. These findings align with theoretical predictions, indicating a good correspondence between experimental results and established theories.

## 1. Introduction

Liquid Crystal Elastomers (LCEs) are unique materials that combine rubber-like elasticity with liquid crystalline properties. Like traditional liquid crystals, their orientational properties are described by a unit vector along the preferred direction of LC molecules called the director. These anisotropic polymers were first theorized by de Gennes in 1975 to possess remarkable responsive abilities [1] and were later actualized by Finkelmann et al. in 1981 [2]. LCEs are particularly noted for their ability to reversibly change shape in response to various stimuli, including heat, light, humidity, and electric fields [3,4,5], a feature that has led to their exploration in actuator applications [5,6,7,8,9]. These distinctive characteristics also make them promising candidates for optical applications [10,11]. Specifically, LCEs are ideal for creating optical devices that are mechanically switchable and tunable, using the anisotropy and programmability of elastomers to achieve controlled deformation. Such devices, which are operable through mechanical actions like stretching, bending, or twisting, allow for precise manipulation of optical properties. Despite the considerable potential of LCEs in this field, the fabrication of devices that are mechanically controlled using LCEs poses significant challenges.

The initial challenge in developing nematic LCEs was synthesizing samples that are homogeneously aligned (monodomain), transparent in the optical range, and operable at room temperature (the glass transition temperature is below the ambient temperature). Over the nearly forty years since LCEs were first developed, a diverse range of chemistries has been explored, with a significant focus on polysiloxane and acrylate-based LCEs [12,13]. Building on this knowledge and by adapting the LCE technology first described by Urayama et al. in 2005 [14], macroscopic room-temperature, transparent LCE samples have been produced, employing acrylate chemistry, as detailed in [15]. These LCEs have also exhibited intriguing mechanical properties, such as an auxetic response at strains > 0.4 [15,16], making them highly promising for a range of applications. Specifically, Mistry et al. [15,16] were the first to report a negative Poisson ratio in liquid crystalline materials. Subsequent detailed studies revealed that in these systems, the nematic director deforms via the mechanism often termed the mechanical Frèedericksz transition (MFT), which is, in fact, mechanically induced nematic biaxiality [15,17]. This is in contrast to the continuous in-plane, uniaxial rotation typically associated with the semi-soft elastic (SSE) response observed in most LCEs [18,19]. The findings of Raistrick et al. and Wang et al. [17,20] confirm that the auxetic response in LCEs is due to out-of-plane rotations of mesogenic units, leading to the formation of biaxial order.

Auxetic LCEs are promising materials for application in areas such as impact resistance, but an important challenge to consider in their development is the complete understanding of the mechanical characteristics of nematic LCEs, even at relatively low strains, which is crucial for their effective design and integration into devices. Different approaches have been proposed for describing the elastic behavior of LCEs, as discussed in the literature [19,21,22,23,24]. Since nematic LCEs are typically regarded as hyperelastic and transversely isotropic, five independent elastic constants are required to fully describe their linear elastic properties [1,19]. Traditionally, the mechanical properties and anisotropy in LCEs are characterized macroscopically by techniques like tensile testing [23,25] and dynamic mechanical analysis [26,27]. However, these methods primarily provide insights into only one or two elastic constants. To determine all five independent elastic constants for nematic LCEs, static mechanical testing in three distinct experimental configurations was performed in this study. As detailed in [28], such an experimental setup enables the direct measurement of three orthogonal strains in response to an applied stress while also assuming that director rotations under strain are negligible.

Mistry et al. [25] calculated the longitudinal and transverse Young’s moduli for an acrylate-based LCE with a higher crosslink density than that studied here. The measurement of the Young’s moduli and Poisson ratios was reported in [28] for acrylate–amine-based nematic elastomers with nematic order parameters varying between 0.33 and 0.43. Furthermore, in [29], calculation of five elasticity coefficients (representing the elastic free energy density of the material) was performed for acrylate–amine-based LCE samples based on the experimental data reported in [28]. Even so, in many studies, liquid crystal elastomers have been characterized by a soft elastic response associated with semi-soft elasticity (SSE), where director rotation accommodates deformation with minimal energy cost. However, recent evidence indicates that some types of LCEs—particularly those with side-chain architectures and light crosslinking—do not follow the SSE deformation mode. Instead, they exhibit a biaxial elastic response, suggesting more complex mechanical behavior than traditionally recognized. Notably, quantitative measurements of the elasticity of these types of LCEs are extremely scarce in the literature. In this context, our study provides one of the first detailed experimental investigations into the uniaxial elastic response of these LCEs at low strain. In this paper, using the elastic free energy density expression from [29] and completing tensile tests for the necessary arrangements, elastic characterization of an acrylate-based LCE sample was performed at low strain; this material has an auxetic threshold at strains of ~0.4 [17]. The monodomain sample has a high nematic uniaxial order parameter, taking values of ~0.6 at the low strains considered in this work [17]. The main elastic constants of these types of LCEs were measured, and for the first time, five elasticity coefficients of the elastic free energy density of these materials were determined.

## 2. Materials and Methods

### 2.1. LCE Sample Fabrication

**Figure 1:**
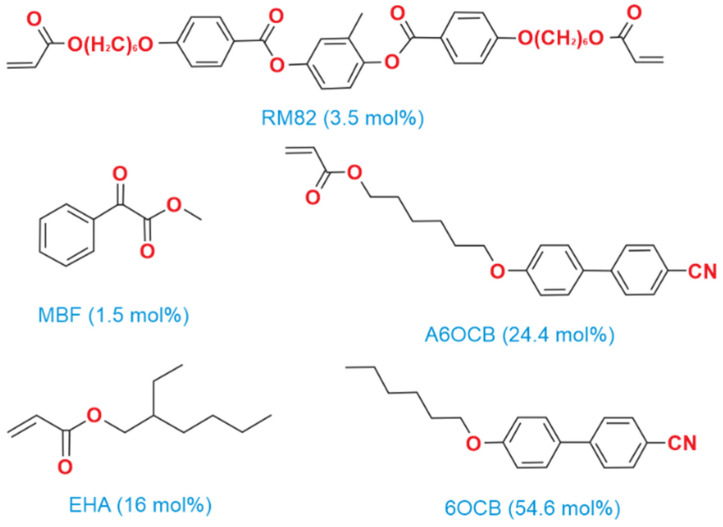
The chemical structures of components involved in the synthesis of acrylate-based LCEs. The 6OCB is not present in the final LCE film.

Acrylate-based LCE samples were synthesized using the methodology outlined by Mistry et al. [15]. The process utilized to prepare the nematic elastomer samples is detailed in [17]. Figure 1 illustrates the chemical compounds mixed in specified proportions to create the nematic LCE precursor. The inclusion of 4′-hexyloxybiphenyl (6OCB), a nonreactive mesogen, extends the nematic phase range of the precursor. The monofunctional reactive compound, 6-(4-cyanobiphenyl-4′-yloxy)hexyl acrylate (A6OCB), forms the mesogenic side groups of the LCE, while 1,4-bis-[4-(6-acryloyloxyhexyloxy)benzoyloxy]-2-methylbenzene (RM82), a bifunctional mesogenic crosslinker, contributes to the network structure. To enhance the flexibility of the polymer backbone and lower the glass transition temperature (below room temperature), 2-ethylhexyl acrylate (EHA) was incorporated. Methyl benzoylformate (MBF) served as the UV photoinitiator. A6OCB, 6OCB, and RM82 were purchased from Synthon Chemicals GmbH, Wolfen, Germany. EHA and MBF were procured from Sigma Aldrich (Gillingham, UK).

The mesogenic components were thoroughly mixed by heating them to 120 °C and stirring at 200 rpm on a magnetic hot plate for 5 min. Following this, the temperature was reduced to 35 °C, at which point 2-ethylhexyl acrylate (EHA) and methyl benzoylformate (MBF) were added, with stirring continued for an additional 2 min. The fabrication of planar samples followed previously described methods [15,16], using 100 µm thick Melinex401 film (DuPont Teijin Films, Redcar, UK) as spacers and glass with Melinex401 polymer as substrates for the LCE mold. To achieve good monodomain alignment, the inner surfaces of the substrates were spin-coated with a 0.5 wt.% polyvinyl alcohol (PVA) solution, which was then uniaxially rubbed after drying. The mixture (at 35 °C, isotropic phase) was introduced into the mold through capillary action and left to cool to room temperature over approximately 20 min. This cooling process allowed the mesogens to align within the nematic phase, in response to the alignment layers. Following alignment, the molds were exposed to a low-intensity UV light source (2.5 mW/cm^2^) for 2 h to initiate curing. After curing, the polymer substrate was delicately removed. Submerging the exposed sample, positioned on the glass substrate, in methanol led to a slight swelling and delamination at the edges. The sample was then fully peeled off using flat-tipped tweezers. Subsequently, any remaining unreacted 6OCB in the LCE was eliminated by immersing it in a dichloromethane (DCM) solution (30% in methanol) overnight. Following the washing step, the LCE film was suspended in a beaker and allowed to dry at 60 °C for 2 h. The glass transition of the sample was 6°C; details of additional characterization of the fabricated samples can be found in [30].

**Figure 2:**
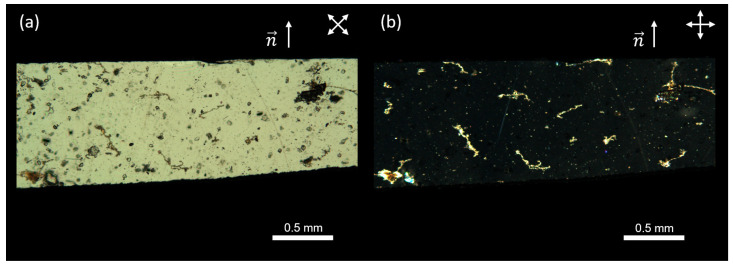
Polarizing microscopy images of the LCE sample obtained using a Leica DM6 M microscope (Leica Microsystems GmbH, Wetzlar, Germany) with crossed polarizers. The top part of each image shows the LCE, and the bottom part serves as a reference with only crossed polarizers. The images illustrate the planar alignment of the sample with the director (**a**) 45° relative to the polarizers and (**b**) 0° relative to one of the polarizers.

**Figure 3:**
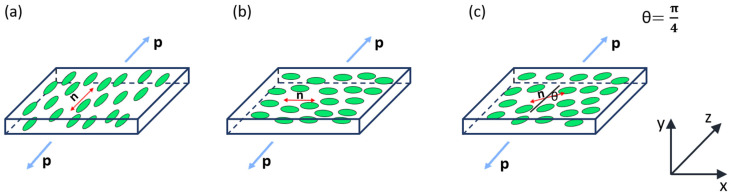
Schematic illustration of three tensile experiments: (**a**) Forces are applied parallel to the nematic director along the z-axis. (**b**) Forces are applied perpendicular to the nematic director along the z-axis. (**c**) The nematic liquid crystal (NLC) director forms a 45-degree angle with the z-axis (loading direction). The applied force per unit area is represented by ‘p’.

For the proposed experiments, two square films, each measuring approximately 10 × 10 mm, were cut from prepared nematic elastomer samples. The first film had a nematic director oriented along the side of the square (See Figure 2 for a demonstration of the high-quality monodomain alignment and uniformity of the sample) and was stretched both parallel and perpendicular to the director (Figure 3a,b). The second film had a nematic director positioned at a 45° angle relative to the stretching direction (Figure 3c). It is worth noting that only geometries where the strain has a component perpendicular to the director can show an auxetic response [16,17,20].

### 2.2. Tensile Experiments

**Figure 4:**
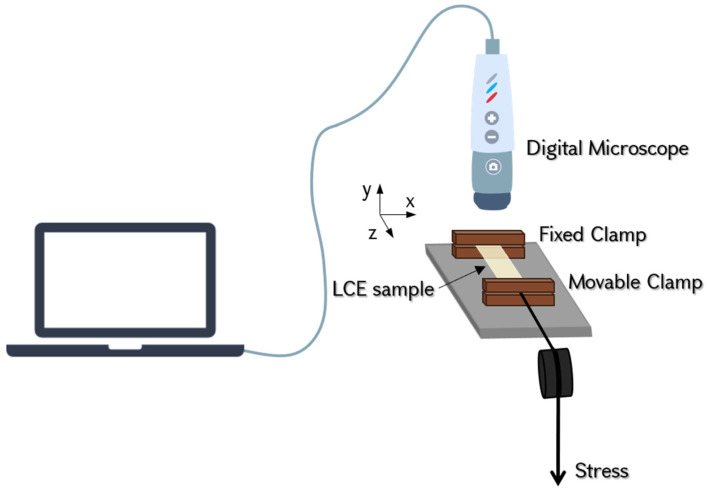
Schematic representation of the experimental setup. The sample is secured at its one edge by a fixed clamp and at its opposite edge by a moveable clamp. The setup enables tensile tests with a maximum load of 3 N (accuracy: 0.2 mN) and can accommodate samples with maximum dimensions of 15 mm × 25 mm (measurement accuracy: 20 µm). The z-axis corresponds to the direction of the applied tensile force (longitudinal direction), while the x- and y-axes represent the lateral and thickness directions, respectively.

The experiments were conducted using a custom-built device (refer to Figure 4) designed to measure the three normal strain components, in contrast to conventional tensile test equipment, which typically captures stress–strain only in the loading direction. The square-shaped film (with dimensions of approximately 10 mm in length, 10 mm in width, and 0.11 mm in thickness) was secured at its opposite ends using fixed and movable clamps mounted on a rail. The clamps were cut using a CO laser, then polished to achieve a flat surface, ensuring even pressure distribution on the elastomer. The setup was built on a metal rail, which enabled direct stretching of the samples, effectively preventing lateral movement of the edges due to shear strains. Stress was generated using a variable weight, which was suspended on a string passing through a pulley and connected to the movable clamp. The images of the sample were captured using a “Prima Expert” digital microscope from Lomo.

Given that LCE samples exhibit viscoelastic properties with dynamic responses to loading, the experimental methodology employs different loading rates to analyze the material’s behavior. For each step, the load was maintained at a constant level for a predetermined period (around 30 s)—long enough to conclude that the fast response regime [28] has ended. However, it should be noted that the stabilization in a steady-state period is much longer. Following this period, the strain is measured to assess the material’s behavior under the specific applied stress. All experiments were conducted at room temperature. It is worth noting that the elastomers were examined with a microscope before the experiment, immediately afterward, and periodically thereafter to assess any signs of degradation. As a result, we did not observe any degradation or changes in the samples.

Parallel to the mechanical loading, strain evaluation was carried out by capturing images of the sample (30 s after stress application) using a digital microscope. The components of the extensional strain in the xz-plane, defined as elongation or shortening per unit length in the corresponding direction, were determined by analyzing captured images with ImageJ software (Version 1.54f). The sample’s initial thickness was precisely measured using a high-accuracy digital micrometer screw gauge and found to be approximately 110 µm. The strain in the thickness direction (y-axis) was calculated by assuming volume conservation, a condition known to be characteristic of these auxetic liquid crystal elastomer samples, as detailed in [16,25]. All experiments were in the low-strain regime (<0.1), below the strain necessary to observe an auxetic response (negative Poisson ratio), which is ~0.4 for this LCE. The assumption of a uniaxial system is further justified, as Wang et al. [17] demonstrated through conoscopy that an unstrained LCE was uniaxial and that it became clearly biaxial at strains higher than ~0.2.

## 3. Results and Discussion

Similar to reports in [28] for acrylate–amine-based main-chain elastomers, the acrylate-based samples in the current study were also found to be viscoelastic. Therefore, preliminary experiments were conducted, varying the strain rate to determine the optimal value, as well as the most suitable timing for conducting experiments within the specified setup. This was necessary because at higher strain rates, viscoelastic materials tend to exhibit more elastic behavior, as the viscous components do not have sufficient time to respond. Conversely, at lower strain rates, the viscous characteristics become more pronounced. This leads to more significant time-dependent deformation, manifesting as phenomena like creep or stress relaxation.

Temperature is another crucial parameter for characterizing materials like these. In this series of experiments, the tests were conducted at ambient temperature, which is ~10 K above the glass transition temperature in this system. Future publications will focus on characterizing the temperature dependency of elasticity coefficients for those LCE samples, as well as exploring their thermomechanical responses.

In all experiments, the measured strains were kept within the low-strain range (0–10% strain), where they should remain within the linear elasticity regime [15].

**Figure 5:**
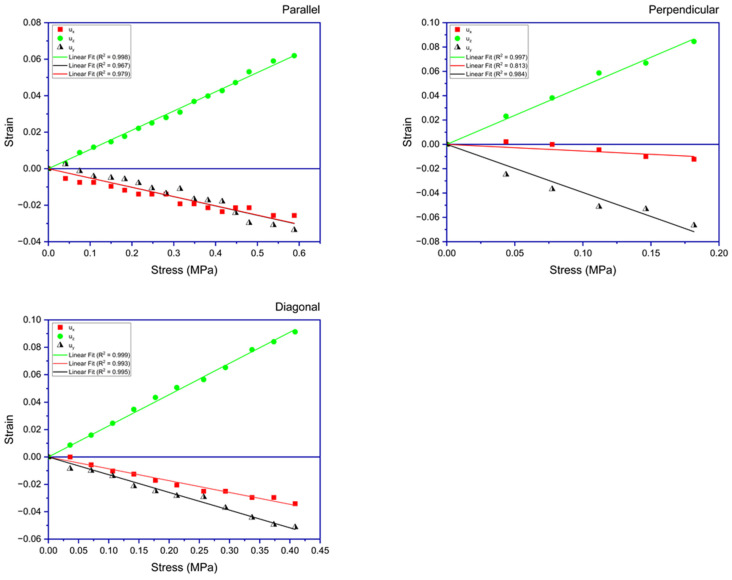
This figure illustrates the strains experienced by the sample when subjected to stress applied in three orientations: parallel, perpendicular, and diagonal to the director. Displayed in each subfigure are the plots of normal strains along the three dimensions of the sample. The depicted lines are the best fits to the obtained data.

Figure 5 presents the stress–strain data for an LCE sample that was subjected to three tensile testing experiments.

Table 1 summarizes the main elastic constants calculated for acrylate-based LCE samples using the stress–strain data. The Poisson ratios for the stretching parallel to the director, $\sigma_{∥y}$ and $\sigma_{∥x}$, for the sample are 0.486 and 0.48, respectively, both nearing 0.5, suggesting uniaxial symmetry in the samples. The Poisson ratios for the stretching perpendicular to the director, $\sigma_{⊥y}$ and $\sigma_{⊥x}$, are observed as 0.831 and 0.112, respectively, indicating a more pronounced response in the transverse plane compared to the axial direction. The value of 0.831 is in good agreement with the Poisson ratio reported in [17], which was around 1.

**Figure 6:**
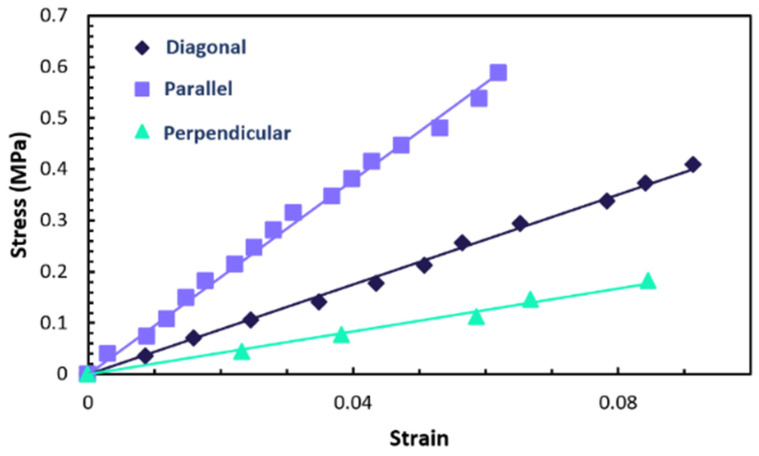
Illustration of the measured stress–strain curves of the sample when stretched in directions parallel, perpendicular, and diagonal to the director. The curves are presented to highlight the variations in Young’s moduli in these different orientations.

To calculate Young’s moduli, fitting of the stress–strain curves was carried out based on proportionality. These curves are collectively displayed in Figure 6 for comparative analysis. The slope of each line in these fits represents the respective Young’s modulus. Specifically, the longitudinal Young’s modulus ($E_{L}$) of the sample is recorded at 9.48 MPa, while the transverse Young’s modulus ($E_{T}$) is noted as 2.096 MPa. These values are close to those reported by Mistry et al. in [25] for samples fabricated through similar chemistry. Additionally, they are close to those found in another type of LCE (acrylate–amine-based main-chain LCEs) and corroborate existing literature data referenced in [28,31,32].

The findings of this study also verify the reciprocal relationship expressed as $\sigma_{⊥x}$/$E_{T}$ = $\sigma_{∥y}$/$E_{L}$, a characteristic of transversely isotropic materials. This consistency in the data underscores the intrinsic symmetry-based properties of the materials under study.

Furthermore, the five elasticity ($\lambda_{i}$) coefficients entering in the free energy density expression of these materials (referenced in Equation (A1)) were also determined. This calculation was based on experimental data and specific relations outlined in Equations (A2), (A4), and (A6). Table 2 displays the values of these five elasticity coefficients.

When comparing the results with previously calculated values for acrylate–amine-based main-chain LCEs [29], it is noted that the obtained values for the five coefficients are higher in acrylate–amine-based LCEs. This difference can likely be attributed to the stronger coupling between the liquid crystal (LC) units and the polymer backbone in main-chain LCEs. In these materials, the LC units are directly integrated into the polymer backbone, unlike in other configurations. This stronger interaction or linkage in the main-chain LCEs affects the material’s properties, leading to higher elasticity coefficients.

## 4. Conclusions

In summary, this study provides a full elastic characterization of acrylate-based liquid crystal elastomers (LCEs), offering some insights into their mechanical behaviors. The main elastic constants for these materials are reported here. Using tensile testing data from three experiments and linking normal strains with elasticity coefficients, the Young’s modulus, Poisson ratios, and the five elasticity coefficients that enter in the elastic free energy density of these materials were determined for the first time. In particular, the longitudinal Young’s modulus of the studied sample was obtained as 9.48 MPa, while the transverse Young’s modulus was obtained as 2.096 MPa, yielding an anisotropy ratio of approximately 4.5. Additionally, when the sample was stretched in the longitudinal direction (parallel to the director), the Poisson ratios obtained in the two orthogonal directions were 0.486 and 0.48, both approaching 0.5, confirming the uniaxial elastic response of these samples at low strain. These findings align with the theoretical predictions associated with the materials’ inherent symmetries, indicating good correspondence between experimental results and established theories. However, the discussed approach is valid only for a low-strain regime (linear response regime). The results reveal that the $\lambda_{i}$ coefficients for these acrylate-based LCEs are lower than for acrylate–amine-based LCEs, as compared with previous calculations. This could be attributed to a stronger coupling between the LC units and the polymer backbone in main-chain LCEs.

Looking ahead, this research can contribute to more complex and precise investigations of LCEs, potentially contributing their application in cutting-edge technologies and devices.

## Figures and Tables

**Table 1:** Main elastic constants determined for acrylate-based LCE sample.

$E_{L}$ (MPa)	$E_{45}$ (MPa)	$E_{T}$ (MPa)	$\sigma_{∥y}$	$\sigma_{∥x}$	$\sigma_{⊥y}$	$\sigma_{⊥x}$
9.48	4.39	2.096	0.486	0.48	0.831	0.112

**Table 2:** Elasticity coefficients for acrylate-based LCE sample.

$\lambda_{0}$ (MPa)	$\lambda_{1}$ (MPa)	$\lambda_{2}$ (MPa)	$\lambda_{3}$ (MPa)	$\lambda_{4}$ (MPa)
0.572	15.042	−0.11	−0.052	8.23

## Data Availability

Data are contained within the article. Further inquiries can be directed to the corresponding author.

## Appendix A

### Determining Elastic Constants for Nematic LCE

Weakly cross-linked nematic elastomers with side chains are considered here, i.e., the case in which the relevant dynamic macroscopic variables can be determined. In contrast, for main-chain nematic elastomers, these variables cannot be defined as straightforwardly because the elastic and nematic contributions cannot be as clearly separated. A uniaxial approximation is adopted, accounting for the inherent anisotropy of the elastomer, resulting in only five independent terms in the purely mechanical component of the strain-free energy. Purely orientational interactions among liquid crystal molecules are considered by incorporating Frank’s strain energy. Finally, the third component of the free energy describes the coupling between mechanical and orientational deformations. The total free energy density of a deformed monodomain nematic elastomer can be expressed as follows [19,29,33]:

(A1) $$\begin{array}{l} F_{tot}=\lambda_{0}{\left(s_{ik}\right)}^{2}+\frac{1}{2}\lambda_{1}{\left(s_{ii}\right)}^{2}+2\lambda_{2}n_{k}s_{ip}s_{kp}+\lambda_{3}n_{i}n_{k}s_{ik}s_{pp}+\frac{1}{2}\lambda_{4}n_{i}n_{k}n_{l}n_{m}s_{ik}s_{lm}+\\ n_{m}\left(e_{kpm}\omega_{p}-a_{km}\right)\left[\frac{1}{2}D_{1}\left(e_{kqr}\omega_{q}-a_{kr}\right)n_{r}+D_{2}n_{i}s_{ik}\right]+\frac{1}{2}K_{1}{\left(divn\right)}^{2}+\frac{1}{2}K_{2}{\left(ncurln\right)}^{2}+\\ \frac{1}{2}K_{3}{\left(n\times curln\right)}^{2} \end{array}$$

where $s_{ik}=\frac{1}{2}\left(u_{ik}+u_{ki}\right)$ represents the symmetric part of the strain tensor; u is the displacement vector, where $u_{ik}=\frac{\partial u_{i}}{\partial x_{k}}$; n is a unit vector called the nematic director; $a_{ik}=\frac{1}{2}\left(u_{ik}-u_{ki}\right)$ is the antisymmetric part of the strain tensor; and ω describes the rotation of the director, defined by δn=ω×n, where $e_{ijk}$ is the totally antisymmetric Levi–Civita tensor. The Frank elastic constants are denoted by $K_{i}$. Here, D1 and D2 represent the free-energy contributions associated with the relative rotation of the director and its coupling to mechanical deformation. Based on symmetry considerations, the energy from these rotations comprises two distinct contributions. The first, D1, describes the free energy associated with the relative rotation between the director (n) and the strain-free network of the elastomer. The second, D2, accounts for the coupling between the network strain and the relative rotations, providing the driving force for the director’s rotation.

To derive the five elasticity coefficients ($\lambda_{i}$) in the free energy density expression (A1), three tensile experiments are discussed (Figure 3).

In the first experiment, the symmetry axis and the nematic director are positioned along the z-axis, with $n_{x}=n_{y}=0$ and $n_{z}=1$, as shown in Figure 3a.

In this particular case, explicit expressions linking the $\lambda_{i}$ coefficients with the experimentally measured normal strains were derived by utilizing the total stress tensor expression derived via differentiating A1 by the strain tensor [29]:

(A2) $$\left\{\begin{array}{c} u_{xx}^{∥}=u_{yy}^{∥}\\ 2\left(\lambda_{1}+\lambda_{0}\right)u_{yy}^{∥}+\left(\lambda_{1}+\lambda_{3}\right)u_{zz}^{∥}=0\\ \left(\lambda_{1}+\lambda_{3}\right)u_{xx}^{∥}+\left(\lambda_{1}+\lambda_{3}\right)u_{yy}^{∥}+\left(2\lambda_{0}+\lambda_{1}+4\lambda_{2}+\lambda_{4}+2\lambda_{3}\right)u_{zz}^{∥}=p \end{array}\right.$$

Regarding the primary elastic constants (Young’s modulus and Poisson ratios) for the first geometry, the following can be obtained:

(A3) $$E_{∥}=\frac{p}{u_{zz}^{∥}}=\frac{\Lambda_{∥}}{\left(\lambda_{0}+\lambda_{1}\right)},\;\sigma_{∥}=-\frac{u_{xx}^{∥}}{u_{zz}^{∥}}=-\frac{u_{yy}^{∥}}{u_{zz}^{∥}}=\frac{\lambda_{1}+\lambda_{3}}{2(\lambda_{0}+\lambda_{1})},$$

where $\Lambda_{∥}=2\lambda_{0}^{2}+3\lambda_{0}\lambda_{1}+4\lambda_{0}\lambda_{2}{+2\lambda_{0}\lambda }_{3}+{\lambda_{0}\lambda }_{4}+4\lambda_{2}\lambda_{1}+\lambda_{4}\lambda_{1}-\lambda_{3}^{2}$.

In the second experiment, forces are applied perpendicular to the nematic director, with $n_{z}=n_{y}=0$, $n_{x}=1$, as illustrated in Figure 3b.

In this scenario, the following connections can be similarly established:

(A4) $$\begin{array}{c} \left(2\lambda_{0}+\lambda_{1}+4\lambda_{2}+\lambda_{4}+2\lambda_{3}\right)u_{xx}^{⊥}+\left(\lambda_{1}+\lambda_{3}\right)u_{yy}^{⊥}+\left(\lambda_{1}+\lambda_{3}\right)u_{zz}^{⊥}=0\\ \left(\lambda_{3}+\lambda_{1}\right)u_{xx}^{⊥}+\left(2\lambda_{0}+\lambda_{1}\right)u_{yy}^{⊥}+\lambda_{1}u_{zz}^{⊥}=0\\ \left(\lambda_{3}+\lambda_{1}\right)u_{xx}^{⊥}+\lambda_{1}u_{yy}^{⊥}+\left(2\lambda_{0}+\lambda_{1}\right)u_{zz}^{⊥}=p \end{array}$$

In this case, the primary elastic constants for the second geometry are determined using a similar approach. Specifically, for Young’s modulus, we obtain:

(A5) $$E_{⊥}=\frac{4\lambda_{0}\Lambda_{∥}}{\Lambda_{⊥}},$$

and the Poisson ratios are

(A6) $$\sigma_{⊥x}=-\frac{u_{xx}^{⊥}}{u_{zz}^{⊥}}=\frac{{2\lambda }_{0}\left(\lambda_{1}+\lambda_{3}\right)}{\Lambda_{⊥}},\;\sigma_{⊥y}=-\frac{u_{yy}^{⊥}}{u_{zz}^{⊥}}=\frac{2\Lambda_{∥}-\Lambda_{⊥}}{\Lambda_{⊥}},$$

where $\Lambda_{⊥}=4\lambda_{0}^{2}+4\lambda_{0}\lambda_{1}+8\lambda_{0}\lambda_{2}+4\lambda_{0}\lambda_{3}+{2\lambda_{0}\lambda }_{4}+4\lambda_{1}\lambda_{2}+{\lambda_{1}\lambda }_{4}-\lambda_{3}^{2}$.

In the third experiment, the nematic director forms a 45° angle with the loading direction, with $n_{y}=0$ and $n_{x}=n_{z}=\frac{1}{\sqrt{2}}$, as shown in Figure 3c.

For the third geometry, the following relations can be obtained:

(A7) $$\left\{\begin{array}{c} \left(u_{xx}^{45}+u_{zz}^{45}\right)S_{0}+u_{yy}^{45}S=0.5\lambda_{3}p\\ \left(u_{xx}^{45}+u_{zz}^{45}\right)S_{3}+u_{yy}^{45}S_{4}=0 \end{array}\right.$$

where $S_{0}=2\lambda_{0}{(\lambda }_{3}+\lambda_{1}),S=2\lambda_{0}\left(\lambda_{1}+2\lambda_{0}-\lambda_{3}\right),S_{3}=0.5\lambda_{3}\lambda_{3}-0.5\lambda_{1}\left(4\lambda_{0}+4\lambda_{2}+\lambda_{4}\right)\lambda_{0}\lambda_{3},S_{4}=0.5\lambda_{3}^{2}-\left(0.5\lambda_{1}+\lambda_{0}\right)\left(4\lambda_{0}+4\lambda_{2}+\lambda_{4}\right)$.

Therefore, based on normal strain measurements and using these expressions, one can determine the five elasticity coefficients in free energy Expression (A1).
